# Stakeholders views of medicines administration by pharmacy technicians on mental health inpatient wards

**DOI:** 10.1007/s11096-019-00880-w

**Published:** 2019-07-18

**Authors:** Joanne Woodward, Alison MacKinnon, Richard Neil Keers

**Affiliations:** 1 School of Health Sciences, The University of Manchester, Oxford Road, Manchester, M13 9PT UK; 2 North West Boroughs Healthcare NHS Foundation Trust, Hollins Park Hospital, Hollins Lane, Warrington, Cheshire, WA2 8WA UK; 3 Greater Manchester Primary Care Patient Safety Translational Research Centre (GM PSTRC), Greater Manchester Mental Health NHS Foundation Trust, Manchester, UK

**Keywords:** Medicines administration, Mental health, Pharmacy technician, Qualitative research, United Kingdom

## Abstract

*Background* The involvement of pharmacy technicians in medicines administration has been highlighted as an opportunity to enhance medicines management support for nurses and service users. Currently, there is no published evidence around this development within psychiatry. *Objective* To explore the perceptions of key stakeholders toward the feasibility and acceptability of pharmacy technician-led medicines administration within a mental health inpatient setting. *Setting* Ten acute adult and older-adult wards across five inpatient units within one UK mental health provider. *Method* Stratified purposeful sampling was used to recruit participants from primary (pharmacy technician, nurse and service user) and secondary (pharmacist, doctor and senior manager) stakeholder groups. One-to-one, semi-structured interviews were audio recorded, transcribed and analysed thematically using Framework analysis. *Main Outcome Measure* Themes arising from perspectives of stakeholders concerning the feasibility and acceptability of pharmacy technician-led medicines administration. *Results* Twenty participants were recruited, including twelve primary stakeholders. Attitudes towards implementation were favourable overall. Anticipated risks included de-skilling of nurses around medicines and a potentially detrimental impact on the nurse-patient therapeutic relationship; these were contrasted by potential benefits including the release of nurse time and medicines education opportunities with staff and service users. *Conclusion* Technician-led medicines administration was perceived as a feasible service, potentially bringing opportunities for medicines optimisation and released nursing time to care. These findings may be a source of guidance for policymakers and researchers who wish to explore the development of such services. Further exploration of safety and effectiveness is required, particularly within mental health settings.

## Impact on Practice

- Pharmacy technician-led medicines administration may be considered as a feasible and acceptable service development for mental health inpatient wards in the United Kingdom (UK).
- It is important that organisations who wish to develop pharmacy technician medicines administration services consider potential risks, benefits, barriers and facilitators; and how these may be measured or evaluated, prior to implementation.
- A summary list of considerations in relation to implementation and outcome measures has been developed, which may benefit policymakers and senior managers that wish to implement or evaluate pharmacy technician medicines administration services.

## Introduction

Nurses spend up to 40% of their workday administering medication [1], and as many people with mental health problems also have co-morbidities such as diabetes, cardiovascular disease and substance misuse [2–4], their medication regimes can be complex. Staff stress, high workloads and busy wards with numerous interruptions are all implicated in medication administration errors on mental health wards [5–8]. Furthermore, there are additional considerations relating to medicines which may be unique to psychiatry or have a higher profile than in general hospitals; including compulsory treatment under Mental Health legislation, covert administration of medicines, rapid tranquilisation, non-adherence to prescribed medication and drug-seeking behaviour [9–12]. Whilst staff may face challenges managing stressful medicines administration rounds alongside competing demands; opportunities for service users to thoroughly discuss their medication regime and any potential side-effects or problems may be overlooked, leading to increased service user anxiety relating to medicines taking, a risk factor for non-adherence [13, 14].

In England, National Health Service (NHS) mental health services are under pressure [15], with 15% fewer mental health nurses working on inpatient wards in 2014 than in 2009 [16]. The use of section powers under the Mental Health Act (1983) [17] continues to rise [18], and many mental health Trusts are operating above maximum bed occupancy levels, leaving wards overcrowded and understaffed [19]. Patient deaths on inpatient wards have been linked with staffing issues such as shortages of experienced nursing staff and high turnover rates [15, 20].

The development and expansion of clinical pharmacy services has been high on the United Kingdom (UK) government agenda for the last two decades. Pharmacy staff are acknowledged as having an important role in optimising medicines use to reduce risk and increase cost effectiveness; [1] policy initiatives now focus on utilising their skills and integrating pharmaceutical care into frontline services [21–24]. For UK Pharmacy Technicians (PTs) in both primary (community pharmacy and General Practitioner surgeries), and secondary care (hospital and specialist service) settings, their role has evolved from that of dispenser to performing more clinical, ward-based or patient-facing tasks such as medicines reconciliation, counselling on new medicines, conducting physical health assessments (such as blood tests, urinalysis, spirometry and blood pressure readings), performing medication brand switches, advising on local prescribing guidelines and final accuracy checking of dispensed medications [25–27]. This role expansion into clinical care was assisted by the introduction of mandatory registration of British (English, Welsh and Scottish) PTs with a regulatory body, the General Pharmaceutical Council (GPhC), in 2007. In order to register, PTs must undertake 2 years consecutive work experience and complete knowledge and competency based qualifications at level 3 on the relevant National Qualifications Framework, from an approved training provider.

As registered practitioners in Britain, there is potential for PTs to administer medicines in their own right; and the shift into patient-facing roles has highlighted the development of clinical skills which could support them in undertaking medicines administration. The same right naturally extends to pharmacists, however the focus for pharmacists has shifted towards non-medical prescribing [28] as opposed to medicines administration. Pharmacy assistants could potentially reduce nursing burden by assisting with medicines administration [29], but as unregistered and unregulated practitioners they are unlikely to have the clinical skills or necessary governance arrangements required to undertake this role alone.

There is an absence of published evidence describing PT involvement in medicines administration in psychiatry. Prior to this research, a literature review was undertaken by the researcher (JW), using a systematic search and retrieval strategy. Published studies have arisen from general hospitals across the United States (US) and UK, and are service development/case-study projects, where single organisations implemented and evaluated services based on their own requirements and priorities. Papers from the US, published during the 1970s and 80s involved a large university hospital, where PTs were employed on general wards for the sole purpose of administering medicines. Numerous papers describing and evaluating various aspects of the service were published [30–36]. However these models of working came to be considered expensive and outdated [37]. One 12 month UK study where PTs administered morning medicines on a 34 bed medical ward, noted a reduction in medication administration errors with one technician error compared to seven nurse errors. However, data were only collected from ten medicines administration rounds within the 12 month period; making it too limited to perform statistical analysis. A further study involving PT supported medicines administration in one English NHS hospital, found that nurses were supportive of the scheme. However, PTs reported they did not always directly accompany nurses on the medicines round, due to feelings that their presence was potentially intrusive [38, 39]. Published discourse relating to PT-led medicines administration within the UK has been mainly within policy documents [21, 23, 27] and there is an absence of evidence to guide health managers on where and how PT-led medicines administration could or should be implemented, the likely acceptability of this to existing stakeholders, and any anticipated benefits or risks. There is also no current published evidence around PT-led medicines administration in mental health services. For this reason, research is needed to explore the views of mental health stakeholders such as nurses and service users toward the feasibility and acceptability of PTs involvement in medicines administration. Such research could be utilised by managers and policymakers who may be interested in future implementation of these schemes.

## Aim of the study

The aim of this research was to explore the perceptions of key stakeholders toward the theoretical feasibility and acceptability of introducing PT-led medicines administration within a mental health inpatient setting.

## Ethics approval

This study was approved by the NHS Health Research Authority and the South Central Oxford-B Research Ethics Committee (Ref 17/SC/0442). Informed consent was obtained from all individual participants included in the study.

## Method

### Design

The study was a qualitative design comprising of one-to-one semi-structured interviews. The research methodology utilised was that of naturalistic inquiry, considered to be suitable for pharmacy practice research due to its practical, less philosophical approach which prioritises the research aim and accurate representation of participant’s views over the philosophical standpoint of the author [40].

### Sample and setting

The setting was ten acute adult and older-adult wards set across five inpatient units within one NHS mental health provider in Northern England. Identified stakeholders included a primary sample of those who would be directly involved in any future delivery of PT-led medicines administration (pharmacy technicians, nurses and service users), and a secondary sample of those likely to be influential in or affected by any future service redesign (pharmacists, doctors and senior managers). Eligibility criteria for the study were: English speaking participants, aged 18 or over, having capacity to provide informed consent and permanent employment on the ward (staff) or current or recent (within 6 months) admission to the ward (service users). A stratified purposeful sampling method [41] was used to select participants recruited from each stakeholder group, to ensure adequate representation. A sample size of 20–25 participants was agreed, with final numbers being determined by the principles of data saturation [42]. Participants were recruited via poster and social media advertising and researcher attendance at ward staff and service user meetings to discuss the research.

### Topic guide development

The topic guide for the interviews was developed with input from the researcher (JW), clinical mentor (AM) academic supervisor (RNK) and patient and public involvement representatives from the NHS Trust. The topic guide focussed on risks and benefits of PT-led medicines administration, barriers and facilitators to implementation, anticipated training requirements and governance arrangements.

### Data generation

Data generation took place from November 2017 to March 2018. Interviews were conducted face-to-face with the participant and a trained researcher (JW), on NHS Trust premises. Written informed consent was obtained from all participants, which included the digital audio recording of interviews. These were then transcribed verbatim by the researcher (JW). The mean interview time was 29 min (range = 17–42 min). All identifiable information were removed during transcription.

### Data analysis

The transcribed data was coded using NVivo™ qualitative data analysis software [43], following the principles of structural coding [44]. This method was chosen as structural codes are based on elements of the interview questions; in this instance risks, benefits, barriers and facilitators. These codes informed the identification of themes and the overall perception of feasibility and acceptability. Open coding was also applied to other emerging themes; such as training, skills and governance.

The coded data was then analysed using Framework analysis [45]. Framework analysis is a common approach for interpretation of semi-structured interviews. It has been shown to be useful in multi-disciplinary healthcare research [46] and has been previously utilised in pharmacy practice research [47]. The preliminary themes and codes were utilised to form an analytical framework [48] which was shared with the academic supervisor (RNK) for discussion, as part of a cyclical process of reviewing and refining the categories and codes with the remaining data. Finally, data was charted into a framework matrix which allowed the data from each transcription to be summarised by category and theme [46].

## Results

### Description of sample

Twenty stakeholders were recruited to the study from the following groups: primary stakeholders—pharmacy technician (n = 5), nurse (n = 4), service user (n = 3); and secondary stakeholders—pharmacist (n = 3), senior manager (n = 4) and doctor (n = 1). To ensure participant anonymity, the information from the doctor was included within the senior manager stakeholder group. The backgrounds of the other senior managers were: nurse (n = 2), pharmacist (n = 1) and allied health professional (n = 1). Staff members had spent an average of 7 years in post (range = 6 months to 20 years). Service users had spent an average of 5.5 weeks (range = 2–8 weeks) on the inpatient ward during their most recent admission. One service user was currently admitted on a ward at the time of the interview.

### Summary of categories and themes

Identified data categories and themes have been summarised in a table (Table 1).

**Table 1:** Summary of identified categories and themes

	Risks	Benefits	Barriers	Facilitators
Themes				
Overall Feasibility and Acceptability	Ward environment/personal safety Pharmacy capacity and PT availability	Opportunities for patient medicines education and optimisation Increased staff skill-mix Staff development opportunities	Technology changes (e.g. electronic prescribing and medicines administration system implementation) Cultural and change culture (nurses, service users and pharmacy staff)	Executive-level support Evidence or president from other mental health organisations Good project management and evaluation Staged implementation
Medicines Education	De-skilling of nurses in medicines use	Service user medicines education and optimisation opportunities Opportunities for nurse development (e.g. prescribing)		
Medicines Safety	No difference in risk	Safer medicines administration (error and missed dose reduction)		
Therapeutic Relationships	Loss of nurse-patient therapeutic relationship	Release of nurse time for quality care		Increased PT visibility on wards
Teamwork	Difference in staff skill set (e.g. awareness of patient presentation)	Pharmacy Technician development Peer support	Lack of role understanding or overlapping roles	Promotion of team working Staff involvement and engagement Clarity around role definition for nurses and PTs
Skills and Training		Clinical skills training for PTs ‘Soft-skills’ training for PTs PTs being currently competent, no training needed	Queries over requirement for accuracy checking Pharmacy culture, expansion to patient-facing roles	Formal competency assessment and/or qualifications Standardisation of training for PTs and nurses (same training) Good supervision arrangements
Governance			Lack of current evidence base for mental health	Trust policies and Standard Operating Procedures National guidance or policy Position statements from relevant professional bodies Risk assessment and scoping exercises

### Overall feasibility and acceptability

There was evidence of widespread acceptability of PT-led medicines administration amongst participants. Generally, the service concept was viewed as an opportunity to develop skill-mix capability and to enhance medicines-related education and support to staff and service users, as described by one senior manager: “I think that swap of knowledge, clinical knowledge, and awareness of the clinical presentation of the patients we’ve got with us, to me it’s like, well, why on earth not?” (Senior Manager 1) Some participants voiced concerns with regards to the cost of running the service along with the capacity of PTs to deliver it: “I’m not particularly against it [PT medicines administration] but it would be on the proviso that there’s backfill for the jobs they were doing.” (Pharmacist 1) yet it was acknowledged that with the right support and planning, a feasibility study could be an opportunity to add to the evidence base around this role: “without that trial period then how are you going to influence the national agenda?” (Senior Manager 1)

### Medicines education

The most frequently identified risk was perceived to be the de-skilling of nurses around medicines. This was particularly apparent within the senior manager group. Specifically, stakeholders reported that PT-led services may reduce exposure to medicines for nurses, which could lessen their everyday knowledge. One senior manager suggested this could potentially limit opportunities for those wishing to pursue advanced practitioner or non-medical prescribing roles: “We’re trying to create non-medical prescribers which will include advanced nurse practitioners, but what you don’t want to do is take away more than you have to of their experience of dealing with drugs on a day-to-day basis.” (Senior Manager 5) However, the risk of de-skilling nursing staff was contrasted by the most important perceived benefit of involving PTs in medicines administration—opportunities for education and training around medicines for nurses and service users. Nurses reported that PTs generally had a more in-depth knowledge of medicines, meaning nurses could learn from working together with PTs: “I think if you look at probably, with regards to especially around contraindications and side effects, I think that a technician would probably know more, have a more advanced knowledge than a registered nurse these days” (Nurse 1)

Some stakeholders raised the possibility of group learning or peer-support sessions on the ward as a way of improving practice around medicines administration:

It would be good for them [PTs] to give formal teaching sessions maybe, to the nurses. Or peer groups with the nurses; monthly professional peer groups, because each group would learn from each other and I think each group brings a slightly different dynamic to the same thing. (Senior Manager 5)

It was highlighted by stakeholders as important to offer opportunities for service users to learn more about their medicines in preparation for discharge. Many stakeholders expressed the view that this could promote informed decision making regarding medicines, and potentially improve adherence. PT2 reflects that PTs may have more opportunities compared to nurses to facilitate medicines education with service users:

If the patient took that opportunity to ask a question, I don’t know whether the nurses would be able to answer at that time and they are so busy, it’s the wrong time sometimes, so I think we’d be able to answer more questions and talk about compliance because you’d have the opportunity to better educate the patients. If they chose not to take their medicines, an informed discussion could take place. (Pharmacy Technician 2)

### Medicines safety

Some stakeholders believed that PTs expertise and focus on medicines would translate to safer medicines administration, primarily due to PTs being interrupted or distracted less than nurses, as well as their ability to detect prescribing errors:

They [pharmacy technicians] are devoted purely and utterly to doing that particular element of the job. So they’re less likely to be interrupted, or have a patient or another nurse or doctor come in and try to accost them because they’re a pharmacy technician. (Nurse 4)

However, the fact that it was mentioned less than medicines education, suggests that participants may not consider differences in medicines safety to be the primary reason for implementation. Furthermore, PTs and pharmacists were more cautious and stated that any medication safety risks would potentially remain the same due to the influence of human factors:

I think the risks would still be the same, [as nurses] you could still give the wrong thing, couldn’t you cos it’s still human approach isn’t it? So, I don’t know whether there would be any more or less, I think the risks would roughly be the same. (Pharmacy Technician 4)

### Therapeutic relationships

Some stakeholders highlighted PT-led medicines administration as potentially having a negative impact on the therapeutic relationship between the nurse and service user. As one service user participant described, the medicines round was viewed by some nurses as an opportunity to assess a service users’ presentation on a one-to-one basis, which may be lost if medicines were administered by the PT: “… because they [nurses] have quite a lot of other pressures, paperwork and stuff like that, sometimes it’s difficult for them to gain contact time with patients and I think that’s an ideal opportunity, if you like, an ongoing assessment” (Service User 2). However, nurses admitted to feeling under-pressure to complete medicines administration within the required timescale, as a result of both nurses and service users attempting to utilise medicines administration as an opportunity for one-to-one engagement, particularly when discussions involved issues that were not medicines related. “It’s very difficult to get your medicines done within the two hour time limit…. You’ve got to concentrate with no interruptions to get it done.” (Nurse 4) The benefit of using medicines administration as a time for therapeutic interactions was viewed by other stakeholders as being minimal: “I don’t think it’s an interaction that’s particularly therapeutic in that way, it is a task, so I’m not sure that there’d be anything lost through that [having PT-led administration].” (Senior Manager 3)

Most stakeholders reported that relationships between nurses and service users could be improved by relocating non-medicines related patient interactions within the medicines round to other times when nurses could focus their attention exclusively on such matters. This could be facilitated by releasing nurse time though greater PT involvement in medicines administration, refocussing the medicines round back to medicines related discussions. These other activities included administrative duties and therapeutic interventions with service users such as recovery focussed planning, activities, talking therapies and discussions around other social support:

We all know how thin nurses’ time is spread, between documentation, speaking to the patients and doing medication rounds and ward reviews. I think if we were to help out in some way with medicines administration, that takes some responsibility off them where they can better document things that have been discussed in ward rounds, get more involved with patients and spend more time with them, where their time should be spent, in my opinion. (Pharmacy Technician 1)

It was acknowledged that PTs could benefit from opportunities to learn from nurses regarding the clinical presentation of service users, and that involving PTs in medicines administration may enhance the therapeutic relationship between service users and pharmacy technicians:

I think it may aid them in feeling more included as part of a team. And actually that direct involvement I think it’d enhance their, potentially enhance, the therapeutic relationship with our service users. At the moment of course they have contact with our patients, but it’s generally more specific to serious medications the likes of lithium, clozapine, where you know some education is needed about concordance, what the processes are about monitoring. (Nurse 3)

### Teamwork

Some stakeholders suggested an alternative model of collaborative working, where the PT and nurse worked together on medicines administration, created an opportunity to make the most of their complementary skills. Participants across all stakeholder groups suggested that this collaborative approach may give nurses more time to undertake clinical aspects of administration whilst the technician performed the technical preparation of medicines, and would potentially alleviate concerns around de-skilling of nurses, particularly in the early stages of implementation:

You’re using the best skill-sets for both staff members and also it’s not necessarily de-skilling the nurses. If anything it’s skilling the nurses up more, because they’ll learn a bit more about the medicines and vice versa with the technician, they learn a bit more about the physical aspects and the assessment (Pharmacist 1)

## Discussion

This is the first study to prospectively explore the feasibility and acceptability of PT-led medicines administration in mental health; its findings supporting current UK policy direction concerning the transformation of clinical pharmacy services. A PT-led medicines administration service was broadly welcomed by participants, with the majority reporting a positive potential impact. Whilst participants reported potential limitations such as the de-skilling of nurses around medicines and a potential detrimental effect on the nurse-patient therapeutic relationship, these were counterbalanced by contrasting accounts of benefits in terms of medicines education opportunities for both staff and service users and a release of nurse time to provide therapeutic activities and recovery planning.

The primary benefit of PT-led medicines administration was reported by participants as opportunities for service user education around medicines, the enhancement of medicines optimisation and shared decision-making. This differs from the primary focus of many acute trust schemes—medicines safety [39, 49–51], and may be due to differences in patient population within mental health services, where medicines adherence is a global public health concern [11, 52]. A lack of knowledge regarding medicines usage and potential side-effects among service users are known risk factors for non-adherence [53] which can cause relapse of illness, resulting in poor quality of life, reduced efficacy of further treatment, waste of healthcare resources and increased risk of suicide [52].Given this, increased medicines related knowledge and adherence should be considered as a primary outcome measure for any future implementation within mental health.

This study highlights the importance of measuring the impact of PT-led services on nurses’ knowledge and skills. Nurses in this study agreed that working more closely with PTs could possibly increase their medicines-related knowledge, which may help with development into advanced practitioner roles. Most stakeholders agreed that if PT-led medicines administration was implemented, nurses would need to retain involvement in some capacity. This view is supported by published evidence from US hospitals, where PT medicines administration services ended due to the perceived safety implications of nurses becoming de-skilled in medicines administration [35, 37]. Whilst PT roles in UK general hospitals are currently being extended into 7 day working [22], this does not include 24 h ward cover and is not the case for many mental health Trusts, where PTs usually provide ward cover during office hours. Therefore, it is probable that models where some nurse involvement is retained; either by alternating medicines rounds with PTs or by PTs working with nurses to administer, may be preferred over models where PTs took overall responsibility.

The release of nurse time is also highlighted in a recent landmark report by Lord Carter: ‘Making better use of pharmacy staff to review medicines choice and optimise supply and administration of medicines enables trusts to release significant amounts of nursing time back to clinical care’ [23 p67]. However, the degree to which nurse time is released depends on the level of involvement they retain—whilst joint working with a PT may release some time during the medicines round; this would be less than a PT completing a medicines round without nurse involvement. Future research observing such changes could be conducted by way of ethnographic observations of medicines related activities [54].

The results of this research add significantly to current discourse concerning PT-led medicines administration, particularly within mental health. This research could potentially be used by British mental health organisations to fulfil the recommendations of Lord Carter’s report [23], that NHS Trusts identify opportunities for innovative use of pharmacy staff to support medicines administration. Further research is needed which builds upon these findings—to design pilot or feasibility studies. As such, a summary list of considerations has been produced based on the collective accounts of participants (Text box 1). These considerations, alongside any future research, could be used as a baseline guide for service planning and implementation by others who may wish to further explore the role of PTs in medicines administration. This may be timely, as many organisations seek to innovate in how they deliver services.

**Table:** 

**Text Box 1** Summary list of considerations
Carefully consider the service model and level of nurse involvement, in relation to the impact on de/up-skilling of nurses, released nurse time, clinical assessment of service users and cost-effectiveness of the service
Identify the resource required for both feasibility work and potential larger scale roll-out
Consider compatibility of PT working hours with availability to administer medicines without impacting on other duties, such as medicines reconciliation
Be mindful of cultural issues, which may be overcome by engagement and involvement of stakeholders affected by organisational changes
Develop a robust project plan with clearly defined roles and outcome measures, with involvement from stakeholders, overseen by a multidisciplinary project group
Develop a communications and engagement plan for stakeholder consultation or feedback
Involve senior leaders from the organisation to champion the project
Ensure governance and indemnity issues are in place to protect staff and service users
Implement a robust training pathway for PTs, with possible alignment to the nurse training pathway
Consider PT time spent on the ward, to develop service user interactions and soft-skills
Consider a phased implementation, starting with regular oral medicines only
**Consideration should be given to including the following as potential outcome measures:**
Evaluation of changes to nurse confidence/competency in medicines administration
Evaluation of release of nurse time, including impact of any therapeutic benefits
Non-inferiority evaluation regarding medicines safety (error rates, missed doses etc.)
Evaluation of medicines knowledge, satisfaction and/or adherence for service users
Evaluation of the accuracy of clinical assessment of presentation of service users by PTs
Evaluation of cost-effectiveness; including training costs, recruitment costs, impact of dual working (where relevant) and impact on existing PT workload

The primary strength of this study is that it is the first to qualitatively explore the feasibility and acceptability of PT-led medicines administration; therefore it has value to any organisation who wishes to implement such a scheme. An additional strength is the specific focus on psychiatry, with findings that highlight issues unique to this sector. Given the timeliness of the study in relation to recent UK guidelines, the study has potential value to healthcare leaders and policymakers. Other strengths include involvement of a variety of stakeholders, including service users, to capture the full range of views; the latter as both study participants and in the design of the study protocol.

A limitation of the study is that the stakeholders had no practical experience of PT-led medicines administration, and so factors discussed were perceived (rather than actual). Other limitations included the small sample size and single-organisation design, which limits transferability of the findings. Furthermore, the study involved only adult acute mental health wards, and so cannot be considered transferrable to other settings such as forensic, learning disability or children’s mental health. The results of this research may be of interest to countries other than Great Britain; however caution should be exercised due to international differences in PT training and registration requirements and the impact of local legislation regarding medicines administration.

## Conclusion

This research shows overall acceptability for PT medicines administration on mental health wards. Whilst participants broadly welcomed PT medicines administration as a service model, they raised questions regarding whether PTs should take responsibility for all medicines administration or whether a hybrid system involving both nurses and PTs may be preferable in order to build team-working and facilitate the sharing of knowledge and skills. The findings of this research identify new lines of inquiry for research in this area, and may form a foundation of guidance for health care leaders and researchers who wish to further explore and develop PT-led medicines administration in future.
